# Semi-automated approach for generation of biological networks on drug-induced cholestasis, steatosis, hepatitis, and cirrhosis

**DOI:** 10.1007/s43188-022-00124-6

**Published:** 2022-03-03

**Authors:** Hyun Kil Shin, Oana Florean, Barry Hardy, Tatyana Doktorova, Myung-Gyun Kang

**Affiliations:** 1 Toxicoinformatics Group, Department of Predictive Toxicology, Korea Institute of Toxicology, Daejeon, 34114 Republic of Korea; 2 Human and Environmental Toxicology, University of Science and Technology, Daejeon, 34113 Republic of Korea; 3 Edelweiss Connect GmbH, Hochbergerstrasse 60C, 4057 Basel, Switzerland

**Keywords:** DILI, Drug-induced liver disease, Hepatotoxicity, Computational toxicology

## Abstract

Drug-induced liver injury (DILI) is one of the leading reasons for discontinuation of a new drug development project. Diverse machine learning or deep learning models have been developed to predict DILI. However, these models have not provided an adequate understanding of the mechanisms leading to DILI. The development of safer drugs requires novel computational approaches that enable the prompt understanding of the mechanism of DILI. In this study, the mechanisms leading to the development of cholestasis, steatosis, hepatitis, and cirrhosis were explored using a semi-automated approach for data gathering and associations. Diverse data from ToxCast, Comparative Toxicogenomic Database (CTD), Reactome, and Open TG-GATEs on reference molecules leading to the development of the respective diseases were extracted. The data were used to create biological networks of the four diseases. As expected, the four networks had several common pathways, and a joint DILI network was assembled. Such biological networks could be used in drug discovery to identify possible molecules of concern as they provide a better understanding of the disease-specific key events. The events can be target-tested to provide indications for potential DILI effects.

**Supplementary Information:**

The online version contains supplementary material available at 10.1007/s43188-022-00124-6.

## Introduction

To protect human health and the environment from undesired adverse effects, safety information on the toxic potential of new molecules has to be generated and revised to regulate their use [1]. Safety assessments usually involve a substantial amount of animal experimentation. This is financially burdensome and time-consuming [2], is not always ethically performed, and sometimes, the relevance for humans is questionable. To address these shortcomings, regulatory agencies worldwide are encouraging the implementation of the 3Rs (reduction, refinement, and replacement) of animal experimentation. Among the numerous alternative approaches being developed and tested, those using toxicogenomics or computational toxicology in general are considered especially promising because they will likely facilitate faster hypothesis generation covering data-rich historical sources as input information. This will provide a more detailed and precise understanding of the mechanisms of toxicity.

Drug-induced liver injury (DILI) is one of the common reasons for terminating drug development projects. Approaches for the early detection of such alerts are needed. Numerous machine learning (ML) and deep learning (DL) models have been developed for the detection of DILI and resulting withdrawal of drug candidates in the early phases of development [3]. Although ML or DL models can accurately predict DILI, their interpretability is still an issue for researchers because the process of decision-making by the models is not explainable in most cases [4]. Therefore, a different approach is required to determine the mechanisms of the development of liver disease due to drug-mediated damage. The adverse outcome pathway (AOP) concept partially addresses the issue. The AOP is a formalized, transparent, and quality-controlled way to describe mechanistic information to endpoints for regulatory purposes [5, 6]. However, a potential problem of the AOP is that it requires a lot of time for experts to manually search the literature and identify key events and the links between them. Furthermore, the suggested constructs are linear and are sometimes too simplistic to cover the entire spectrum of events leading to an adverse outcome. Thus, another approach is needed to broaden the understanding of how to deploy knowledge in the development of safer drugs.

Biological networks are one of the most common ways to represent a sequence of molecular and cellular events leading to toxic effects following exogenous exposure [7]. Such networks provide a systemic approach for key events identification and targeted testing by means of alternative approaches [8]. For some complex diseases such as DILI, biological networks are an appropriate way of capturing events and relationships as they cover the entire spectrum of relationships, which can also be non-linear. In evidence collection for biological network development, use of historical data and novel computational approaches for data analysis and assembly have proven very useful for hypothesis generation [9].

In this study, we used a semi-automated approach for diverse data gathering, harmonization, and integration to identify important key events and their relationships in the development of four DILI diseases: cholestasis, steatosis, hepatitis, and cirrhosis. Diverse data from different levels of biological organization extracted from ToxCast, Comparative Toxicogenomics Database (CTD), Reactome, and Open TG-GATEs were subjected to association analysis. Top-ranked genes and pathways associated with the respective diseases were identified and interlinked. As expected, the four studied diseases had a significant number of overlapping biological processes. Finally, a joint DILI network was assembled. Oxidative stress was an obvious major factor contributing to the development of DILI.

## Materials and methods

### Chemical-gene-pathway-disease mapping

Following a previously described approach [9], frequent item set mining methods were applied to high-throughput screening, gene expression, in vivo studies, and disease data present in ToxCast and the CTD. ToxCast provides high-throughput toxicity screening in vitro assay data, and active/inactivity calls on one target gene were collected. CTD provided chemical-gene and chemical-disease interactions. Chemical-gene interactions were defined as activity or expression variation due to chemical treatment, and chemical-disease interactions were inferred associations between chemicals and diseases through genes commonly associated between the chemical and the disease. Reactome database clustered pathways according to function similarity, and pathway hierarchy data was used in the mapping.

### Selection of reference chemicals for the diseases

In this study, cholestasis, steatosis, hepatitis, and cirrhosis were selected as the most common diseases among DILI cases in post-market phase based on PharmaPendium^®^ database (https://www.pharmapendium.com/, accessed Dec. 2018) which curated the post-market reports based on disease terms. After the removal of not-specific adverse endpoints such as liver injury and hepatotoxicity, post market reports for these four DILI cases were more than 10,000 in numbers, respectively. Some drugs were withdrawn from the market due to development of severe DILI even though there were no sign of DILI in preclinical and clinical test, therefore, the diseases were selected based on post-market reports only. A set of reference compounds specific for each disease of interest were selected. Positive reference chemicals induce the disease of interest. Negative chemicals are those for which there is no evidence in the literature of an association to the disease of interest. This is a challenging task as most of the known chemicals coincidently cause several of the studied diseases, rather than being disease-specific. As this was a very important part of the analysis and forms the basis of the developed biological networks, the following strategy was applied. First, search of publicly available literature sources was performed to identify chemicals that were specific to one of the four diseases. Once the disease-specific chemicals had been identified, those also present in Open TG-GATEs [10] were selected for further analysis. The list of OpenTG-GATEs compounds was compared with the list of LiverTox compounds. The LiverTox DB provides up-to-date comprehensive and unbiased information about DILI (https://www.ncbi.nlm.nih.gov/books/NBK547852/). The idea was to obtain a list of compounds that lead to DILI and have available gene expression data. Furthermore, the probable mechanisms of action as listed according to LiverTox were extracted whenever possible or available. The chemicals were split into groups according to the cause of the diseases. Finally, compounds in each group were compared to post-market reports on the four diseases.

### Refinement of chemical-gene-pathway-disease associations

Transcriptomics data from Open TG-GATEs of the selected set of chemicals were applied to customize chemical-gene-pathway-disease associations to the reference chemicals. The fold changes between treatment and control were used from transcriptomics data based on p values. Genes were labeled as toxic group-specific if they showed statistically significant deregulation (− 1 < Log2 fold change > 1 and p value 0.05 by the Welch t test) over more than three chemicals. This approach was applied identically to both positive and negative groups at each time point and dose combination. The genes were further selected only if they were deregulated mostly by the positive reference chemicals. Finally, to ensure that all the identified specific genes were differentially expressed compared to that in response to the negative group of chemicals, the individual gene profiles were manually examined using a bar chart time-series to acknowledge changes in expression over time and by heatmap validation to acknowledge grouping and differential expression in comparison to the negative group of chemicals. This additional curation was done to capture group-specific genes involved in the development of a disease and to remove the individual chemical-specific behaviors. For example, if there is a gene deregulated in response to three chemicals only, it would fulfill the above-described cutoff criteria and would automatically be selected and initially appear in the gene list. This would be a captured chemical-specific behavior, rather than disease-specific. Therefore, an additional step of individual gene examination was performed. Only the group-specific genes were used in the further analysis.

**Fig. 1:**
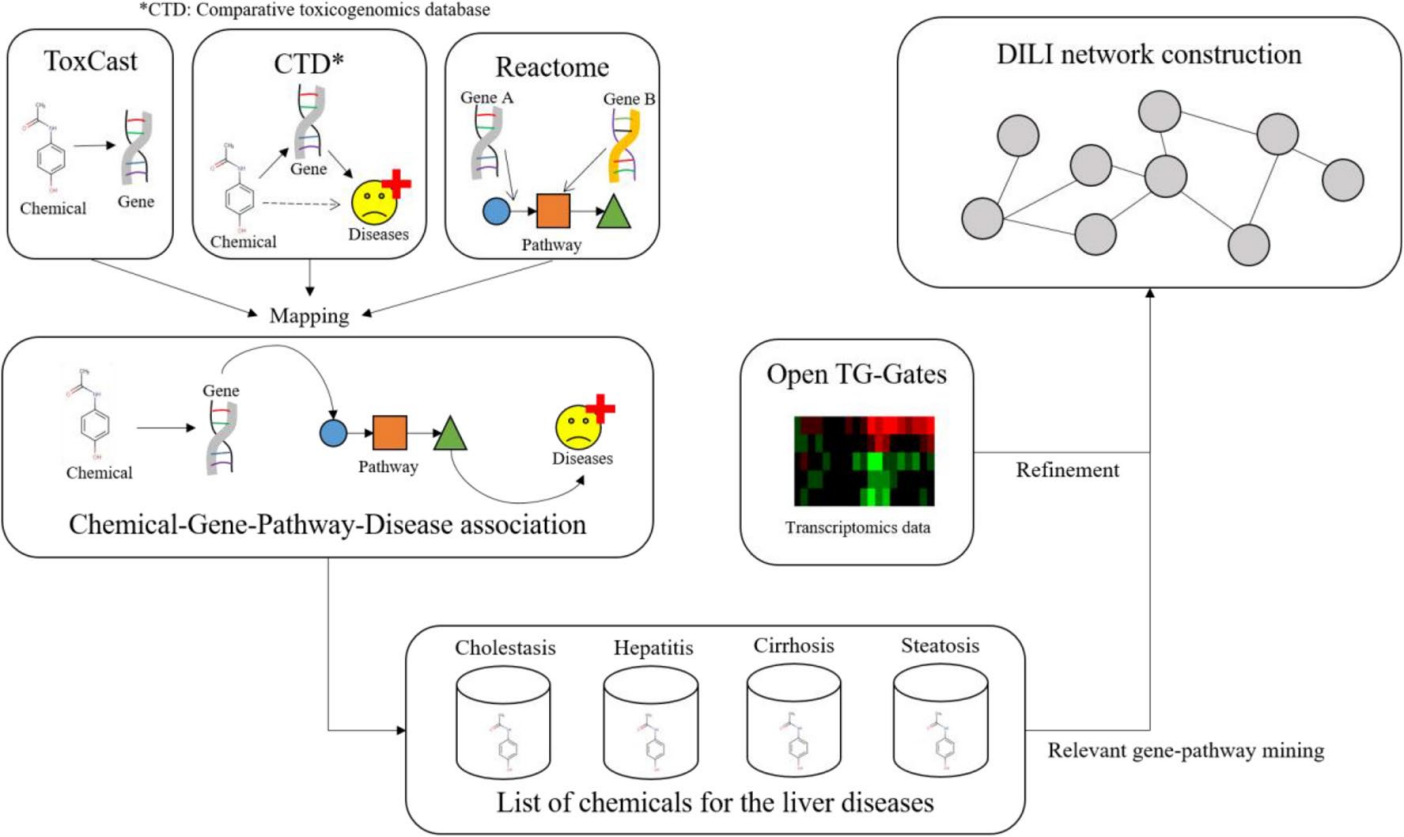
Workflow of this study. Associations between chemicals, genes, pathways, and diseases were established based on data from ToxCast, CTD, and Reactome. This association was further refined with TG-Gates data. Query molecules for each disease were used to extract relevant associations

The goal was to extract associations between genes and diseases that co-occur across datasets. The four diseases were used as input queries, and the output data of the highest probability of a disease of interest to be connected to a set of genes was extracted. Figure 1 describes the workflow of this study.

### Biological networks assembly

Lastly, biological networks were defined as a set of biological events (e.g., at a gene, protein, and pathway level) and relationships assembled. To accomplish this, the first step was to search for direct links between the selected genes. This did not yield very promising results. Thus, the next step was to examine the possibility of interlinking through connector genes. This was done using the GeneMANIA (https://genemania.org/) web-based application for gene function prediction [11]. Once the biological networks were compiled, the processes in which these direct and indirect interacting genes were involved were identified. This was done mostly through a literature search and manual curation.

## Results

### Genes associated with the four liver diseases

**Fig. 2:**
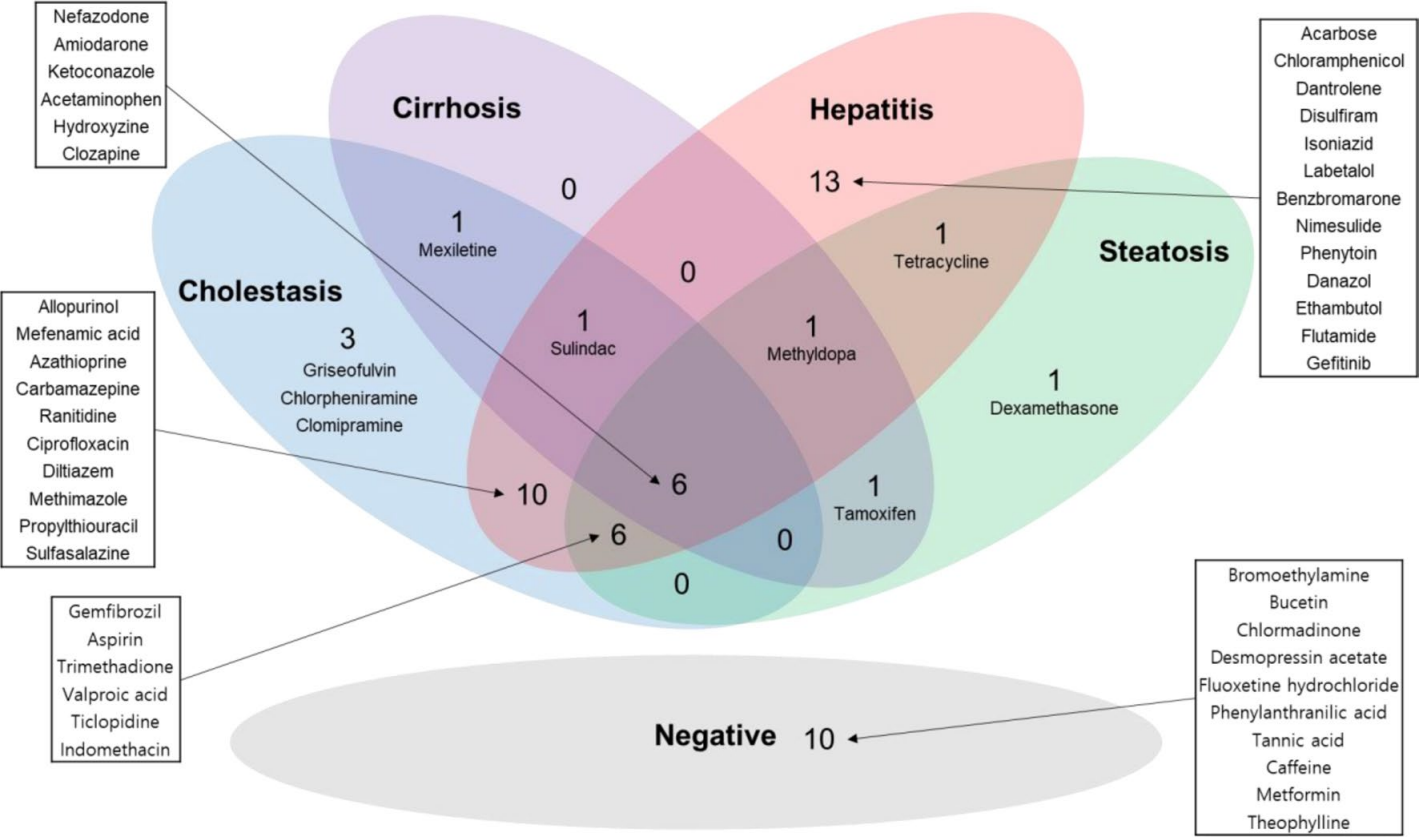
Venn diagram for chemicals selected for each disease. Most of the compounds were labeled over multiple DILI subtypes

**Fig. 3:**
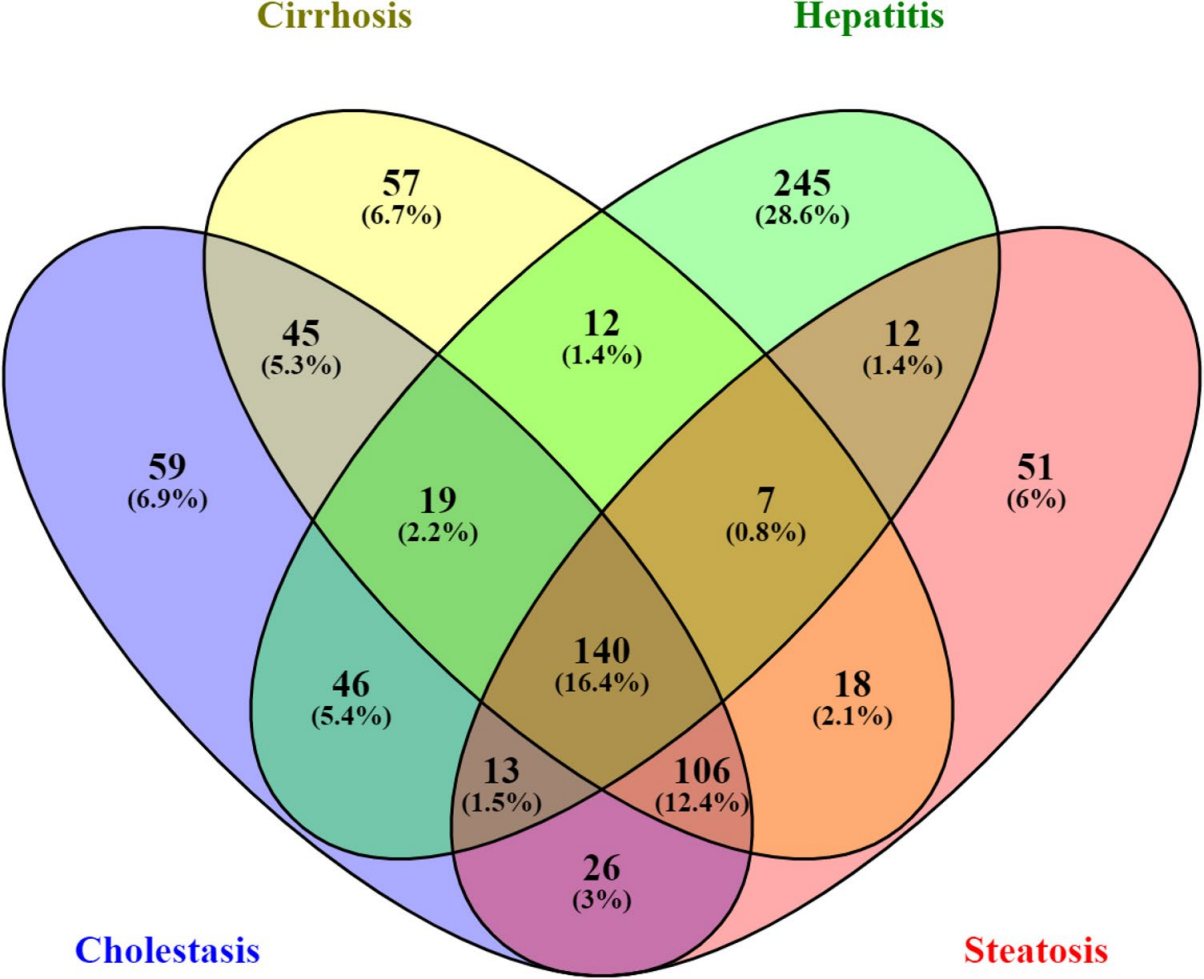
Venn diagram of the genes associated with the diseases

**Fig. 4:**
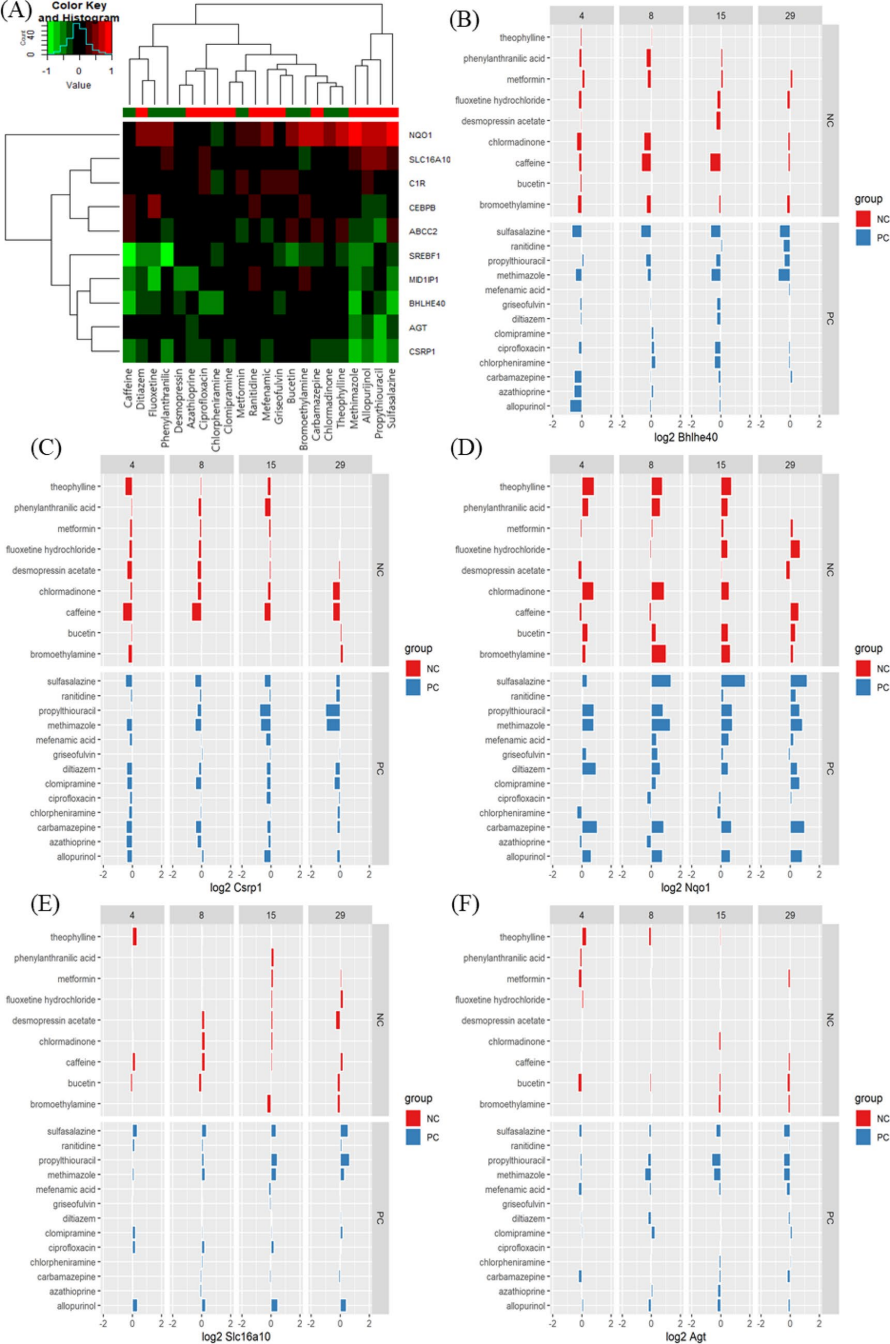
Heat map (**A**) and bar chart (**B–F**) analyses of genes associated with drug-induced cholestasis by comparison between positive compounds (PCs) and negative compounds (NCs). Five genes were consistently deregulated among PCs and displayed a different trend in NCs: BHLHE40 (downregulated, **B**), CSRP1 (downregulated, **C**), NQO1 (upregulated, **D**), SLC15A10 (upregulated, **E**), and AGT (downregulated, **F**)

**Fig. 5:**
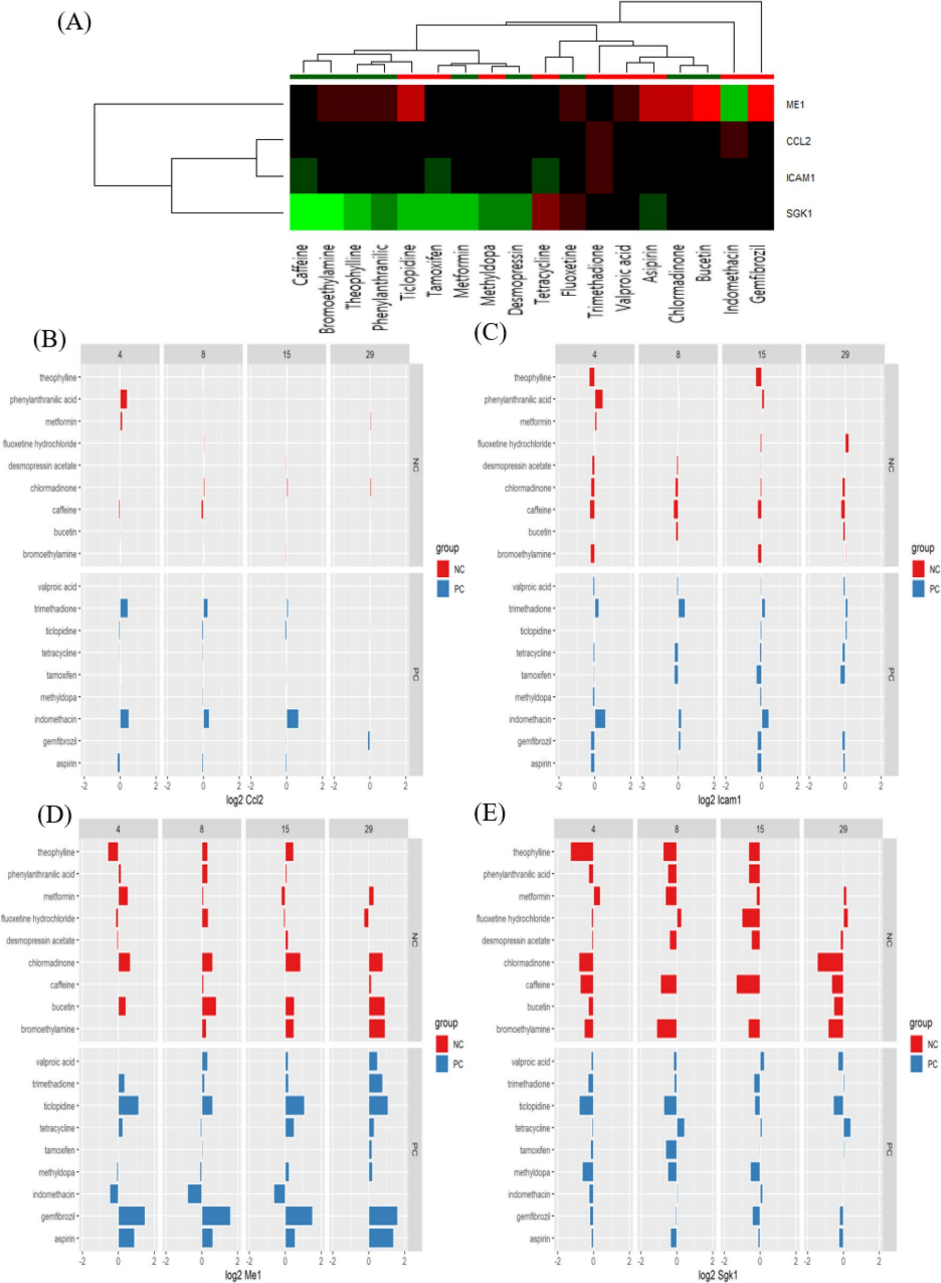
Heat map (**A**) and bar chart (**B**–**E**) analyses of genes associated with drug-induced steatosis by comparison between positive compounds (PCs) and negative compounds (NCs). Four genes were consistently upregulated among PCs and displayed a different trend in NCs: CCL2 (**B**), ICAM1 (**C**), ME1 (**D**), and SGK1 (**E**)

**Fig. 6:**
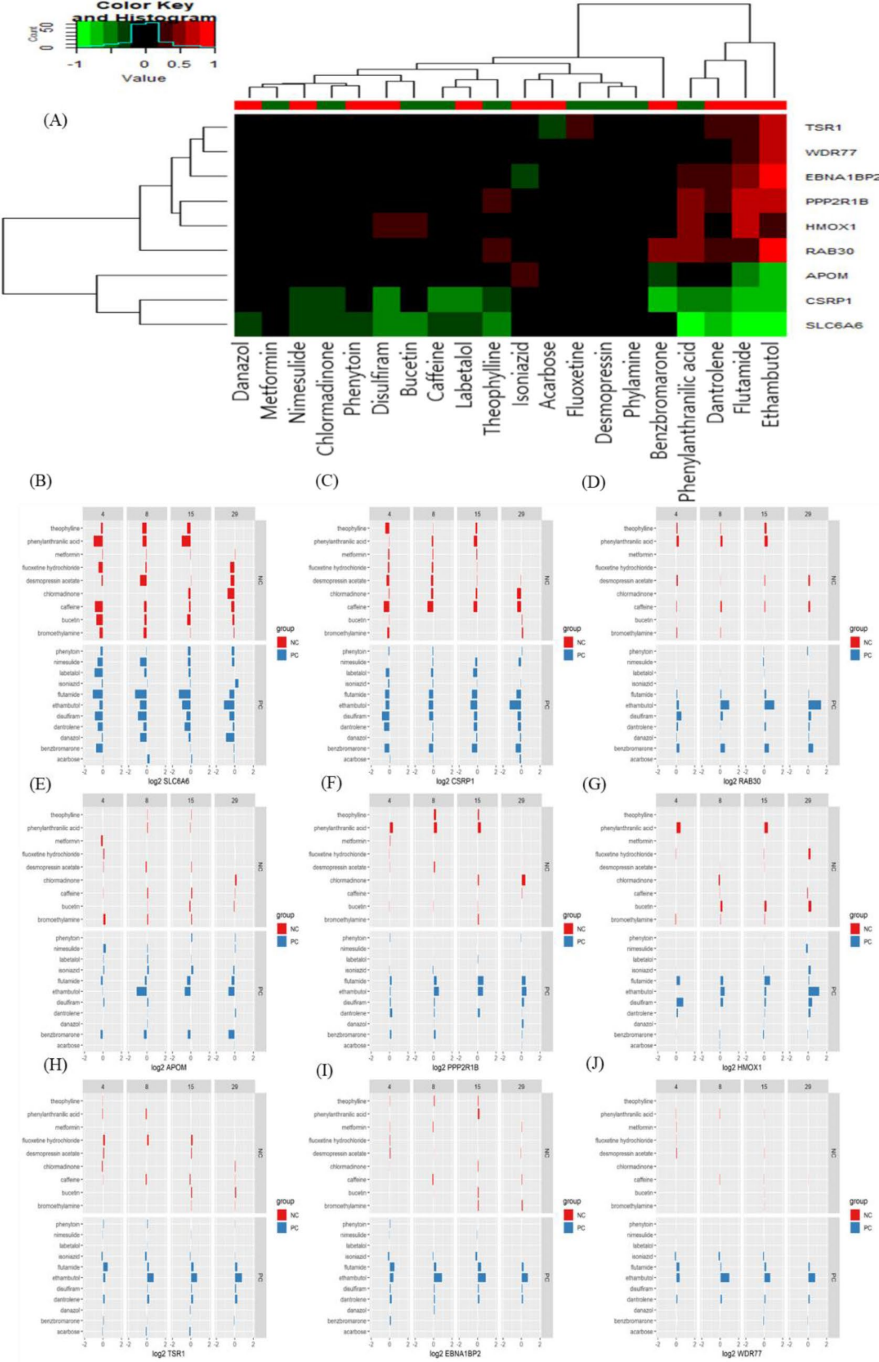
Heat map (**A**) and bar chart (**B**–**J**) analyses of genes associated with drug-induced hepatitis in comparison between positive compounds (PCs) and negative compounds (NCs). Nine genes were consistently deregulated among PCs and displayed a different trend in NCs: SLC6A6 (B), CSRP1 (**C**), RAB30 (**D**), APOM (**E**), PPP2R1B (**F**), HMOX1 (**G**), TSR1 (**H**), EBNA1BP2 (**I**), and WDR77 (**J**)

**Fig. 7:**
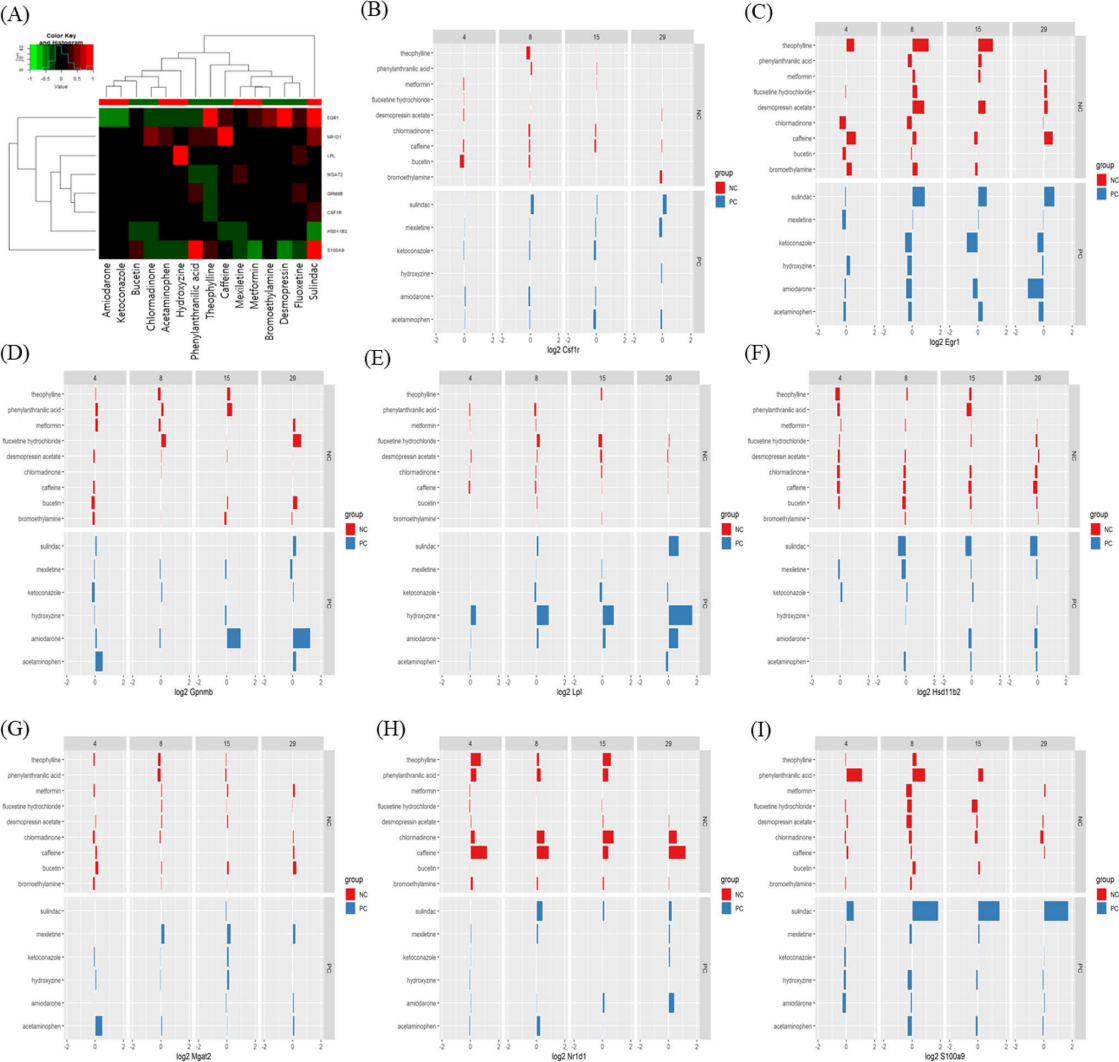
Heat map (**A**) and bar chart (**B**–**I**) analyses of genes associated with drug-induced cirrhosis in comparison between positive compounds (PCs) and negative compounds (NCs). Nine genes were consistently deregulated among PCs and displayed a different trend in NCs: CSF1R (**B**), EGR1 (**C**), GPNMB (**D**), LPL (**E**), HSD1182 (**F**), MGAT2 (**G**), NR1D1 (**H**), and S100A9 (**I**)

The reference chemicals (Fig. 2) were used in chemical-gene-pathway-disease mapping. Querying the assembled chemical-gene-pathway-disease output for the selected disease of interest resulted in 200 to 300 characteristic genes per disease. There was a substantial gene overlap of approximately 140 genes between all four diseases (Fig. 3). The largest difference at the gene level was evident for hepatitis, with 28.6% of the genes specific to hepatitis. The follow-up pathway analysis also indicated a substantial overlap in the number of affected pathways between all four diseases. Thus, a second tier was added. By incorporating data on disease-specific reference chemicals, identification of genes highly specific to the disease of interest was possible. After the application of the second tier, 32 genes highly associated with the development of cholestasis were initially identified (Table 1). The expression of each gene was individually examined and compared to the negative control group to identify whether these genes could serve as biomarkers to characterize and identify cholestasis-specific mechanisms. This further refinement showed that most of the identified genes were also deregulated in a similar fashion in the negative control group, with the exception of five genes (BHLHE40: basic helix-loop-helix family member e40, CSRP1: cysteine and glycine rich protein 1, NQO1: NAD(P)H quinone dehydrogenase 1, SLC16A10: solute carrier family 16 member 10, and AGT: angiotensinogen) that were consistently deregulated in the positive group as opposed to in the negative group (Fig. 4). In the case of steatosis, 20 genes were initially identified as highly associated with its development (Table 2). Further examination revealed that only four genes (CCL2: C-C motif chemokine ligand 2, ICAM1: intracellular adhesion molecule 1, ME1: malic enzyme 1, and SGK1: serum/glucocorticoid regulated kinase 1) were consistently upregulated in the positive group, and they exhibited a different trend in the negative group (Fig. 5). Twenty-five genes were initially identified as being highly associated with the development of hepatitis (Table 3). Further examination revealed that only nine genes (SLC6A6: solute carrier family 6 member 6, CSRP1, RAB30: RAS oncogene family member, APOM: apolipoprotein M, PPP2R1B: protein phosphatase 2 scaffold subunit abeta, HMOX1: heme oxygenase 1, TSR1: ribosome maturation factor, EBNA1BP2: EBNA1 binding protein 2, and WDR77: WD repeat domain 77) were consistently deregulated in the positive reference group, whereas they were unaffected or less affected by the negative reference group of chemicals (Fig. 6). In the case of cirrhosis, 37 genes were initially identified as highly associated with the development of cirrhosis (Table 4). Further examination showed that only eight genes (CSF1R: colony stimulating factor 1 receptor, EGR1: early growth response 1, GPNMB: glycoprotein Nmb, LPL: lipoprotein lipase, HSD11B2: hydroxysteroid 11-beta dehydrogenase 2, MGAT2: alpha-1,6-mannosyl-glycoprotein 2-beta-*N*-acetylglucosaminytransferase, NR1D1: nuclear receptor subfamily 1 group D member 1, and S100A9: S100 calcium binding protein A9) were consistently deregulated (Fig. 7).

**Table 1:** Genes identified as of highest probability to be associated with the development of cholestasis

Time	29Days	15 days	8 days	4 days	1 day	9 h	6 h	3 h
Genes	ABCC2	ABCC2	CD63	A1BG	ABHD3	A1BG	CARS	A1BG
	ADSSL1	ACLY	CYP26B1	ABHD3	CYP26B1	ABHD3	EGR1	ABHD3
	BHLHE40	AGT	CYP7A1	ACLY	EGR1	BHLHE40	HSPB8	IFIT1
	CD63	CSRP1		BHLHE40	MID1IP1	CEBPB	NARS	NQO1
	CFD	CYP27A1		C1R	UHRF1	CREM	S100A9	USP18
	CREM	IFIT1		MID1IP1		CYP7A1		
	CSRP1	IL13RA1		TM7SF2		MID1IP1		
	CYP7A1	S100A9				NQO1		
	IP6K2					SREBF1		
	MID1IP1							
	NQO1							
	PPP2R1B							
	S100A9							
	SLC16A10							
	SREBF1							
	USP18							

**Table 2:** Genes identified as of highest probability to be associated with the development of steatosis

Time	29 days	15 days	8 days	4 days	1 day	9 h	6 h	3 h
Genes	CROT	CROT	A1BG	ME1	ACLY	ACLY	ACLY	INSIG1
	ME1	ME1	CROT	SLC22A8	CCL2	EGR1		SGK1
		S100A9	EGR1		CROT	ME1		
			ME1		EGR1	PHGDH		
			SLC22A8		ICAM1	SREBF1		
					KLKB1			
					LAMC2			
					ME1			
					MYC			
					S100A9			
					SOD2			
					SREBF1			
					TARS			
					USP18			
					XBP1			

**Table 3:** Genes identified as of highest probability to be associated with the development of hepatitis

Time	29 days	15 days	8 days	4 days	1 day	9 h	6 h
Genes	HBB	HBB	SLPI	SLPI	EGR1	EGR1	EGR1
	CROT	SLPI	CROT	CROT	HMOX1	HMOX1	MID1IP1
	FOXA2	EGR1	SLC6A6	CSRP1	MID1IP1	XBP1	RAB30
	SLC6A6	CROT	RAB30	ABHD3	TSR1	MID1IP1	CEBPB
	CSRP1	SLC6A6	TM7SF2	IP6K2	AURKA	RAB30	CROT
	REEP5	CSRP1		SREBF1	RAB30	CYP26B1	
	RAB30	PPP2R1B			CYP26B1	SREBF1	
	APOM	RAB30			DNAJB9		
	SREBF1	CEBPB			EBNA1BP2		
					WDR77		

**Table 4:** Genes identified as of highest probability to be associated with the development of cirrhosis

Time	29 days	15 days	8 days	4 days	1 day	9 h	6 h	3 h
Genes	CSRP1	ABHD3	CROT	C1R	A1BG	ABHD3	CSF1R	CYP26B1
	DUSP6	CSRP1	HSD11B2	DUSP6	CYP26B1	BHLHE40	CYP26B1	NR1D1
	EGR1	CYP27A1	LPL	HBB	CYP27A1	CEBPB	GPNMB	NR1D2
	HBB	EBP	PTPRF		EGR1	CYP26B1		SLC16A10
	LPL	GPNMB			GPNMB	DUSP6		
	PHGDH	HBB			KCNJ8			
	PPP2R1B	LPL			KDR			
	S100A9	NCALD			LAMC2			
		PPP2R1B			LPL			
		S100A9			MGAT2			
					NARS			
					NR3C1			
					NT5E			
					PHLDA1			
					POLR3G			
					SLC1A4			
					TARS			
					WARS			

### Biological network compilation

**Fig. 8:**
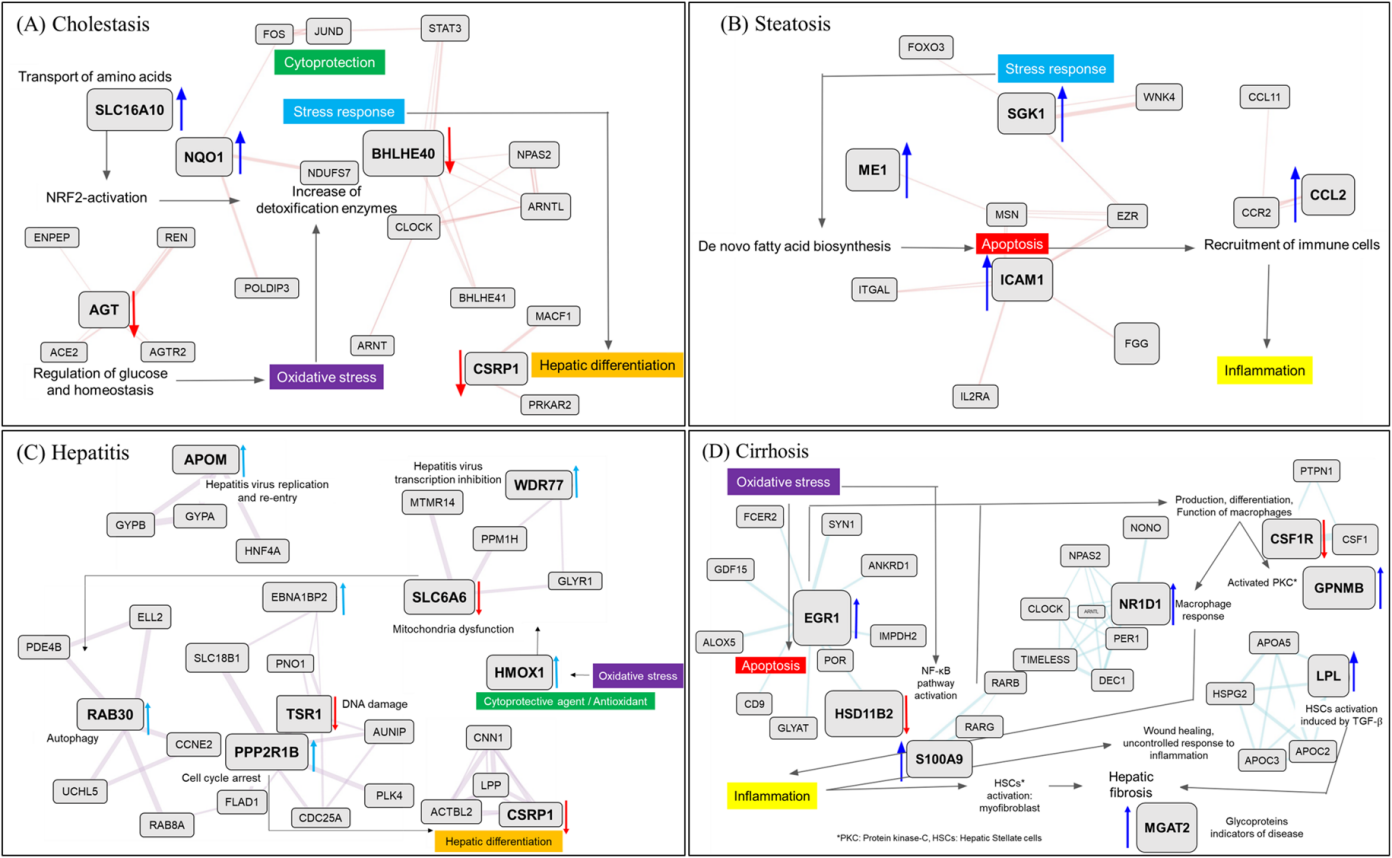
Each biological network for cholestasis (**A**), steatosis (**B**), hepatitis (**C**), and cirrhosis (**D**)

**Fig. 9:**
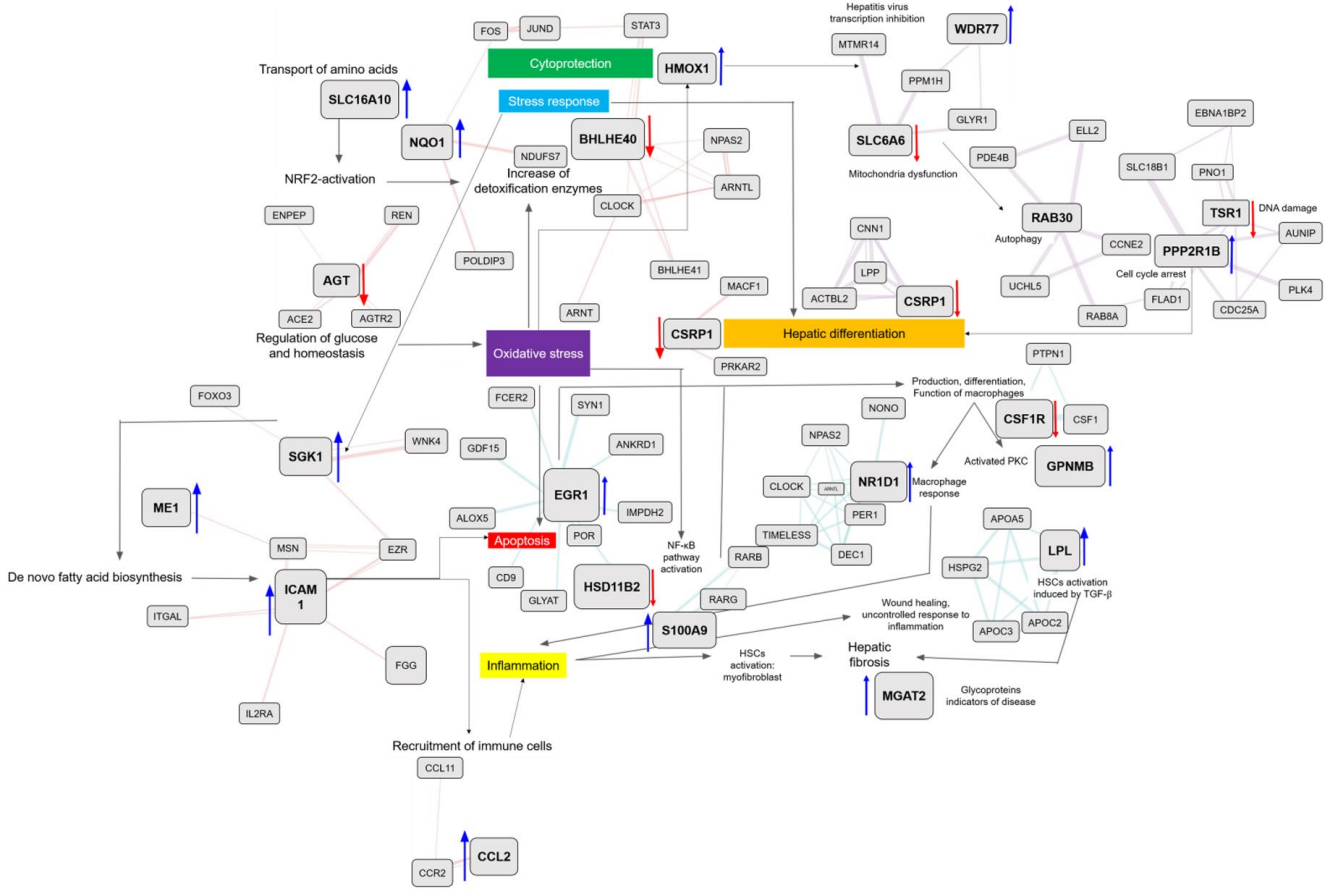
Integrated biological networks on four liver diseases

The identified disease-specific genes were assembled in a network by using the GeneMANIA tool along with manual curation to identify directly and indirectly connected genes. Using this approach, four networks comprising one network each for cholestasis, steatosis, cirrhosis, and hepatitis were generated. In the case of cholestasis (Fig. 8A), the main processes triggering the development of the disease were NRF2 (nuclear factor erythroid 2-related factor 2) activation, oxidative stress, increase in expression of detoxification enzymes, and cytoprotection. The decrease in the level of AGT, which is involved in the regulation of glucose and homeostasis, indirectly leads to an increase in oxidative stress [12]. This increase stimulates the natural defenses of the organism, with increased activities of detoxification enzymes. This is also supported by NRF2 activation, represented by an increase of NQO1 level [13, 14] and increased transport of amino acids by SLC16A10 upregulation [15]. Although the goal of the responses is cytoprotection, a general stress response to the downregulation of BHLHE40 also occurs [16]. This is further reflected in the decrease of responses leading to hepatic differentiation by a decrease in CSRP1 level [17]. The findings indicate that oxidative stress plays an important role in the development of cholestasis. In the steatosis network (Fig. 8B), the main identified triggers were an increase in the stress response, as indicated by an increase in SGK1 level [18, 19]. This can be associated with increased *de novo* fatty acid biosynthesis, as verified by the increase in ME1 level [20–22]. This is linked to an overall increase in apoptosis (upregulation of ICAM1) [23] which leads to the recruitment of immune cells and inflammation via the upregulation of CCL2 [24]. For the hepatitis network (Fig. 8 C), important identified pathways were, again, mainly involved in responses to increased oxidative stress, which initiates cytoprotective action of upregulated HMOX1 [25] leading to mitochondrial dysfunction associated with downregulation of the SLC6A6 taurine transporter [26]. Mitochondrial dysfunction triggers autophagy events by increasing RAB30 level [27] and its linked genes. Increased PPP2R1B level is associated with cell cycle arrest [28] and decreased TSR1 level [29] is associated with DNA damage. Cell cycle arrest causes hepatic differentiation, which maps to the decreased expression of CSRP1 and its linked genes. Upregulated APOM [30] and WDR77 [31] and their associated genes are related to hepatitis virus replication and re-entry. Similarly, in the case of the cirrhosis network (Fig. 8D), the results revealed a central role for oxidative stress. Increased oxidative stress leads to apoptosis associated with upregulated EGR1 [32, 33] and activation of the NF-κB (nuclear factor-kappa B) pathway through the downregulation of HSD11B2 [34, 35]. The genes surrounding EGR1 and HSD11B2 are also important for the mapped events. Activation of apoptosis and the NF-κB pathway leads to production, differentiation, and function of macrophages connected to decreased CSF1R level [36] and its interacting genes. This event promotes the activation of Kupffer cells (evident as increased GPNMB level) [37] and macrophage response (evident as increased NR1D1 level) [38]. The macrophage response causes inflammation (upregulated S100A9) [39], which promotes uncontrolled wound healing and hepatic stellate cell (HSC) activation (evident upregulation of lipoprotein lipase) [40]. HSC activation produces hepatic fibrosis, and glycoproteins appear as indicators of the disease (increased MGAT2 level) [41]. After analysis of each network, the four networks were combined to create a general DILI network to identify overlapping or common events (Fig. 9). Oxidative stress was evident in all four diseases. Cholestasis and hepatitis shared cytoprotection and hepatic differentiation. Cholestasis and steatosis featured a general stress response as the most common process. Steatosis and cirrhosis seem to be involved in apoptosis.

## Discussion

Drug-induced hepatic injury is the most common reason for the termination of drug development projects or withdrawal of market-approved drugs. Characteristic DILI-associated diseases include cholestasis, steatosis, hepatitis, and cirrhosis. Cholestasis is a condition in which the flow of bile from the liver is slowed or blocked. Steatosis features abnormal retention of fat within liver cells. Impaired hepatic lipid storage usually leads to liver metabolic dysfunction and inflammation. Cirrhosis is an irreversible scarring of the liver that leads to impaired liver function. It is considered a terminal stage of chorionic liver impairment. Hepatitis is characterized by general inflammation of the liver, triggered by either chemical exposure or viral infection. Although all these diseases have specific characteristics, they are interconnected in their manifestation. Discrimination of these diseases can be difficult as they can progress from one of the diseases to another. For example, steatosis, cholestasis, and hepatitis can progress to cirrhosis, which is considered an end-stage liver disease. Better understanding of the mechanisms behind the progression of these diseases is important. Many predictive tools have tried to provide clarity using numerous cell-based systems, animal models and *in silico* algorithms. However, progress has been unsatisfactory.

We used semi-automated approaches for data gathering and integration to try to capture the major events responsible for the progression and development of the four selected diseases. The added value of the selected approach includes its fast and robust performance. Diverse data from high-throughput screening, gene expression, *in vivo* observations, and various diseases can be harmonized, analyzed, and assembled to generate the complex DILI biological network. The collective findings indicate that oxidative stress is a central mechanism in the pathogenesis of DILI. Results of the applied semi-automated approach for biological network assembly revealed an increase in the release of reactive oxygen species (ROS) in all four diseases. ROS are associated with an increase in cytoprotective mechanisms and decrease in hepatic differentiation. Furthermore, when such damage is persistent, the natural defenses of the organism are over-ruled and cell damage occurs. This leads to the generation of oxidative stress, mitochondrial dysfunction, and endoplasmic reticulum stress, which may result in scarring and necrosis. At the same time, increased cellular damage stimulates the immune response, whereby inflammasomes trigger an adaptive immune response. Normally, the liver has natural defense mechanisms. However, in DILI, these natural defense mechanisms are compromised, resulting in deteriorated oxidative stress, mitochondrial damage, cell death, and inflammation. These series of events have recently been described in the literature as biomarkers for early identification of DILI in clinical practice [42].

Another important identified mechanism is apoptosis. It seems to be a common event between steatosis and cirrhosis. However, in the case of cirrhosis, the inflammatory response is markedly more pronounced due to the simultaneous action of macrophage response and activation. Cell death is an important mechanism contributing to the development of DILI [43]. Although important, apoptosis is not a central mechanism for DILI development, and therefore, inhibiting apoptosis is insufficient to prevent liver injury. Furthermore, it is not an individual event, but rather an interconnected series of chain events involving increased oxidative stress, refractory increase of cytoprotective mechanisms, and failure of cytoprotection due to the DILI-compromised mechanisms, and eventually mitochondrial dysfunction and apoptosis.

As this approach identified the significant biological events related with the diseases, it can be used to suggest possible molecular initiating events (MIEs), key events (KEs), and key event relationships (KERs) in AOP development. Currently AOP development requires massive manual curation to identify MIEs, KEs, and KERs. Furthermore, AOPs that have been suggested seem to be over-simplified to cover the whole process of adverse outcome development. Therefore, this study can help enrich the understanding of the adverse outcome development. Another possible application of this approach is to suggest biological descriptors and interpret the biological significance of the descriptors. Since hepatotoxicity is generally caused by reactive metabolites rather than drug molecule itself, sometimes molecular descriptors calculated from the parent compounds show a tendency of low correlation with the target endpoint; therefore, most of in silico models used complicated ML or DL algorithms to improve prediction accuracy with the price of renouncing the interpretability of the models. To overcome the limitation of molecular structure-based prediction models, toxicogenomics profiles were used in prediction model. Yen Low et al. used toxicogenomics profiles in hepatotoxicity prediction model development and achieved higher prediction accuracy compared to the model developed with molecular descriptors alone [44]. As toxicogenomics profiles cover wide range of genes, descriptor selection is needed in order to make model building process efficient, and model interpretation also requires analysis of selected descriptors. The approach suggested in this work can be used both in selection and interpretation of the model. As Xi Chen et al. recently developed deep generative adversarial network (GAN) to generate toxicogenomics profile [45], GAN-driven toxicogenomics profile data can be increased, and thus, the semi-automated approach in this study is expected to be applied in wider range of chemicals.

## Conclusions

Semi-automated approaches for data gathering, analysis, and biological network assembly can be valuable as they provide a robust and unbiased way to handle large amounts of diverse data. In this study, the approaches were applied to identify genes deregulated by the reference chemicals that cause the four liver diseases (cholestasis, steatosis, hepatitis, and cirrhosis). Biological networks of each disease was compiled based on the selected genes, and these were combined to deduce significant events in the progression of the four chemical-induced liver diseases. The assembled network can be used to identify important key events and their relationships in the development of four DILI diseases: cholestasis, steatosis, hepatitis, and cirrhosis. Suggested key events are basis for initial testing of chemicals responsible for the development of any of the diseases, and they can be further refined once the patterns of deregulation are consistently recorded.

## Electronic Supplementary Material

Below is the link to the electronic supplementary material.

Supplementary Material 1
